# How to Preserve Cider

**Published:** 1879-09

**Authors:** 


How to Preserve Cider.—A pure sweet cider is only obtainable from clean, sound fruit, and the fruit should therefore be carefully examined and wiped before grinding.

In the press, use hair cloth or gunny in place of straw. As the cider runs from the press let it pass through a hair sieve into a large open vessel that will hold as much juice as can be expressed in one day. In one day, or sometimes less, the pomace will rise to the top, and in a short time grow very thick. When little white bubbles break through it, draw off the liquid through a very small spigot placed about three inches from the bottom, so that the lees may be left behind. The cider must be drawn off into very clean, sweet casks, preferably fresh liquor casks, and closely watched. The moment the white bubbles before mentioned, are perceived rising at the bunghole, rack it again. It is usually necessary to repeat this three times. Then fill up the cask with cider in every respect like that originally contained in it, add a tumbler of warm sweet oil, and bung up tight. For very fine cider it is customary to add at this stage of the process about half a pound of glucose (starch sugar), or a smaller portion of white sugar. The cask should then be allowed to remain in a cool place until the cider has acquired the desired flavor. In the meantime clean barrels for its reception should be prepared, as follows: Some clean strips of rags are dipped in melted sulphur, lighted and turned in the bunghole, and the bung laid loosely on the end of the rag so as to retain the sulphur vapor within the barrel. Then tie up half a pound of mustard seed in a coarse muslin bag, and put it in the barrel, fill the barrel with cider, add about a quarter of a pound of isinglass or fine gelatine dissolved in hot water.

This is the old fashioned way, and will keep cider in the same condition as when it went into the barrel, if kept in a cool place, for a year.

Professional cider makers are now using calcium sulphite (sulphite of lime) instead of mustard and sulphur vapor. It is much more convenient and effectual. To use it, it is simply requisite to add one-eighth to one-quarter of an ounce of the sulphite to each gallon of cider in the cask, first mixing the powder in about a quart of the cider, then pouring it back into the cask and giving the latter a thorough shaking or rolling.

After standing bunged several days to allow the sulphite to exert its full action it may be bottled off.

The sulphurite of lime (which should not be mistaken for the sulphate of lime) is a commercial article, costing about 40 cents a pound by the barrel. It will preserve the sweetness of the cider perfectly, but unless care is taken not to add too much of it, it will impart a slight sulphurous taste to the cider. The bottles and corks used should be perfectly clean, and the corks wired down.

A little cinnamon, winter-green, or sassafras, etc., is often added to to sweet cider in the bottle, together with a drachm or so of carbonate of soda at the moment of driving the stopper. This helps to neutralize free acids, and renders the liquid effervescent when unstopped; but if used in excess it may prejudicially affect the taste.—*Scientific American.*

